# Pilot study: undergraduate sports & exercise medicine conferences: what role do they play?

**DOI:** 10.1136/bmjsem-2020-000787

**Published:** 2020-08-21

**Authors:** Arshan Jimmy Dadrewalla, Hari Venkatesh Pai, Neil Limaye, Rohan Shankarghatta, Shammak Roy-Kundu, Brendan Guest

**Affiliations:** Guy’s Campus, King’s College London Faculty of Life Sciences and Medicine, London, UK

**Keywords:** Education, medicine, sports & exercise medicine

## Abstract

**Objectives:**

Sports & exercise medicine (SEM) is a specialty encompassing the management of medical problems and injuries related to physical activity through means such as exercise advice and prescription. The field of SEM has been recognised in the UK since 2005 yet there is inadequate exposure of SEM in medical curricula. Conferences may be a way to increase exposure where students meet SEM professionals, gaining greater understanding of SEM career pathways. We therefore carried out a pilot study to assess this.

**Methods:**

The King’s College London Sports & Exercise Medicine Society organised a student-led conference consisting of six lectures. Seventy-five delegates were given questionnaires on their views on SEM before and after the conference, assessed using the 5-point Likert scale. Results were analysed using a Wilcoxon-Signed Rank statistical test.

**Results:**

Questionnaire feedback showed 67.4% of delegates (n=46) had received SEM related teaching in their current degree. Results of our statistical analysis showed an increase in SEM career interest (p=0.0359), an increase in understanding of what a career in SEM involves (p=0.0009) and an increase in delegate’s understanding of what is required to pursue a career in SEM (p=0.0004) after our conference.

**Conclusion:**

The study showed issues regarding poor exposure to SEM in medical curricula and highlighted the value of student conferences. Students felt they learnt more about the roles within the SEM specialty, aiding future career progression. Thus, we suggest that student-led conferences are a good platform to bridge this gap while medical schools introduce more SEM into their curricula.

## INTRODUCTION

Sports & exercise medicine (SEM) is a developing area within medicine that includes the medical care in sports and exercise as well as addressing health challenges a population faces through musculoskeletal medicine, exercise advice and prescription.^1^ Labelling the field of SEM as purely the treatment of athletic injuries is a common misconception; the scope of SEM encompasses the therapeutic benefits of physical activity that could benefit both the general population’s physical and mental health.^2^ Though the principles of sports medicine can be traced back to over 5000 years ago, the field of SEM has only been recognised officially as a medical specialty in the UK since February 2005, making it a relatively new career pathway compared with the more established specialties.^3 4^

SEM specialty posts are competitive, with 42 applicants for 14 places to begin SEM training at specialty training level (ST3) in 2019, producing a competition ratio of 3.^5^ There is a lack of in-depth specific teaching within the medical curriculum on SEM compared with other medical specialties.^6^ Studies have found that medical students want greater exposure to SEM content within their curricula compared with what is currently available.^7^ It has been found that only 52% of a British final-year medical student sample group felt comfortable providing physical activity advice, despite SEM concepts being integral within various fields.^8^ Furthermore, with the known lack of integration of SEM within medical school curricula, there is an opportunity for medical conferences to help students and professionals increase their knowledge of the specialty.^9^ Conferences allow for networking with professionals involved within the field, as well as providing opportunities to meet other like-minded students which may increase the likelihood of pursuing it in the future.^10^ Therefore, we felt our study would help gauge and understand the impact of undergraduate conferences on students’ perceptions of the SEM field, based on the student feedback from the conference.

## METHODS

**Figure 1:**
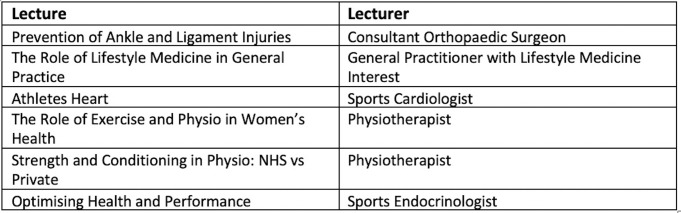
Outline of conference talks.

The Annual Sports & Exercise Medicine Conference ran in November 2019 as a student-led initiative (students from the King’s Sports & Exercise Medicine Society Committee). Our society hosts an annual conference as part of the events plan and in 2018, hosted the British Association of Sports & Exercise Medicine (BASEM) conference. Members of the society agreed that there was a need for increased exposure to SEM in addition to that currently provided. A conference was considered the most appropriate forum to increase student exposure and understanding of what a career in SEM entails. Evidence has shown that conferences are principal sources of educational networking, collaboration and information sharing.^11^ The conference ran as an extra-curricular event which consisted of six lecture talks lasting 40 min each. These were chosen to provide a wide variety of the different roles available in sports medicine and how they are facilitated by different practitioners such as surgeons, GPs, cardiologists, physiotherapists and endocrinologists. An outline of the different talks is provided in figure 1. The conference was open to students from any university, though attendees were primarily studying subjects related to SEM. We chose to run this as a pilot study.

### Feedback

Following the conclusion of the conference, questionnaires were given to all 75 attendees which asked them on their understanding of SEM before and after the conference to measure student exposure and perception of SEM. These questionnaires consisted of statements which were then graded on a 5-point Likert scale: strongly disagree was assigned the score of 1, disagree 2, neither 3, agree 4 and strongly agree 5. A Likert scale was chosen as it allows users to rate the degree to which they agree or disagree with a statement in a quantitative way.^12^ In the questionnaire, students were also encouraged to provide any written feedback regarding the conference.

### Statistical methods

Because we were using Likert scale, our data were qualitative in nature, and it provided non-parametric data. As our data involved the same people, it was classified as paired data, with one categorical and one nominal variable. We decided either to do a two-paired sample test or Wilcoxon-Signed Rank test to validate whether there was a change in the factors measured depending on whether our data were found to be normally distributed. We used the statistical software SPSS Version 24 (IBM SPSS Statistics for Windows, Version 24.0; IBM Corp., Armonk, New York)

From the results of our data, it was clear our data were not normally distributed. Therefore, we decided to do a non-parametric Wilcoxon-Signed Rank test to identify differences in the scores before and after the conference. To see which we had to do we plotted our data on a series of bar charts.

### Patient and public involvement

This study involved no patients and was made up of students attending an undergraduate student-led conference and required no medical or personal information. All participants in this study gave approval for their anonymous data to be used towards potential future research was required from the conference delegates.

## RESULTS

### Demographics

**Figure 2:**
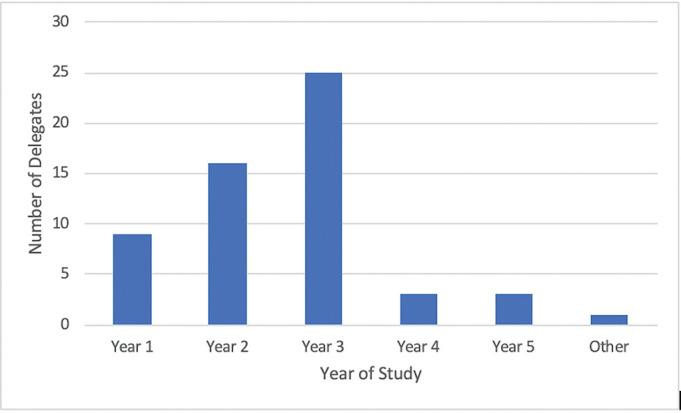
Number of delegates by year of study.

A total of 75 delegates consisting primarily of Year 1 to 3 students (as shown in figure 2) attended the conference, from various universities such as King’s College London, University of Nottingham, University of Plymouth, and among others. The questionnaire was sent to all attending delegates 2 days following the conference.

### Exposure

There were 46 responses to the feedback form sent out by the society to collate information on the exposure of SEM-related teaching in various degrees. 63% of respondents stated that they understood what a career in SEM involves prior to the conference. This figure increased to 80.4% after the conference and the results demonstrate that the respondents’ knowledge of what is required to pursue a career in SEM also developed after the conference; this figure rising from 39.1% before conference to 71.7% after. Moreover, 67.4% of attendees stated they had received SEM-related teaching in their current degree, with 32.6% stating they had not had any at all; 89.1% of respondents stated that they would recommend this conference to a colleague (see online supplemental table 1).

### Statistical analysis

**Figure 3:**
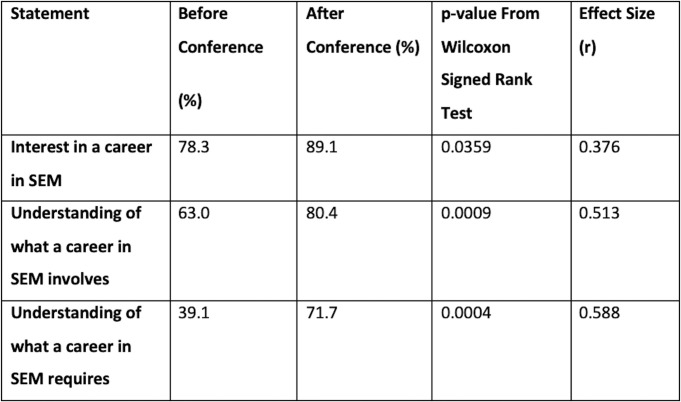
Results from before and after the conference for statements measured. Shown with p value.

From the results of our Wilcoxon-Signed Rank test, there was a statistically significant difference (at 95%) between our delegate scores before and after the conference for the parts of our questionnaire as shown in figure 3.

The significant difference was due to students reporting an increase in these categories. Therefore, we can say that after our conference students reported being more interested in a career in SEM (p=0.0359) and reported having more knowledge about what a career in SEM required (p=0.0004) and involved (p=0.0009). Along with p values, the effect sizes for the results were also calculated to quantify the difference in the categories before and after the conference and are shown in figure 3. These values indicated a small effect (r=0.376) in the difference between students’ increased interest in SEM. A medium effect size was seen for students’ knowledge about a career in SEM involves (r=0.513) and requires (r=0.588).^13^

## DISCUSSION

### Connecting with SEM professionals

Promoting exposure in any medical specialty helps candidates to make informed career choices and permits early stage preparation for entry to competitive training programmes.^10^ Connecting students with senior clinicians provides opportunities for insight into the requirements needed for training entry and the lifestyle involved in the chosen specialty, this was demonstrated by our results showing that students understanding of what a career in SEM involved increased following the conference. Our conference allowed for students to discuss and question SEM clinicians at both a ‘Question and Answer session’ and between lectures. The results of our questionnaires show that our conference improved students’ knowledge about a career in SEM. There was a 17% increase in ‘understanding what a career in SEM involves’ in students after our conference. Our results also demonstrate an increase in clarity of what is required to pursue a career in SEM (see online supplemental table 2).

10.1136/bmjsem-2020-000787.supp1 Supplementary data

### Student conferences

Our results are in line with previous student society conferences.^14^ In 2018, the King’s College London Neurosurgery Society conference was shown to improve knowledge and confidence in students. One study showed that medical students want more exposure to SEM content within their curricula compared with what is currently offered.^6^ From our results, this is evident with only 67% of attendees stating that they had received SEM-related teaching in their current degree and 32.6% stating they had not had any at all. Structured SEM teaching may benefit undergraduate medical students as most students reported an increase in SEM knowledge after the conference (see online supplemental table 3).

### SEM opportunities

For the majority (71.7%) of students at our conference, this was the first SEM conference they had ever attended. From our results, it seems the conference was well received; 89.1% of students agreed or strongly agreed that they would recommend this conference to a colleague and 93.4% agreed or strongly agreed that they were satisfied with the content of the conference. From questionnaire feedback, we were pleased to see that many of the students after the conference were motivated or had better understanding of how to pursue research or a work placement in SEM as shown by our statistically significant results. Both of these factors offer further opportunities to explore the specialty. Future research should investigate the impact of initiatives (such as student conferences) on future SEM applications to determine any objective benefits of these events on the workforce. This could include determining the optimal method of information delivery at such conferences, the effect of practical workshops and ideal conference duration. Research comparing conferences at multiple institutions and involving larger sample sizes would be of great value. Improving the quality of SEM conferences can help fill gaps within university curricula but also help to ensure that future SEM job applicants are better informed and of higher quality.

### Strengths and limitations

A strength of this study is the response rate; of the 75 delegates attending the conference, 46 responded to the questionnaire, giving a response rate of 61.3%. This surpasses the accepted survey response rate of 60%.^15^

There are, however, a number of limitations of this study. As with any survey-based research, the reliability of all data collected is dependent upon the accuracy of answers provided by respondents themselves.^16^ Additionally, there is a risk of response bias; respondents may have provided answers that they perceived would be of interest to the conference organising committee, meaning there is a risk that their answers do not accurately reflect their experiences of the conference.^17^

## CONCLUSION

We reported our experience in establishing a student-led undergraduate SEM conference and presented our structured programme as a potential framework for future student-led conferences. The results of our study demonstrated a lack of exposure to SEM in undergraduate curricula in a cohort of students interested in SEM. Feedback from our cohort indicated students valued the conference and it improved the knowledge of a potential career in SEM and increased their likelihood to pursue a career in SEM.

**Summary box**

**What are the new findings?**

- The main findings of our study were that medical students benefit from increased exposure to sports & exercise medicine through increased awareness of the specialty, pathways and different roles which may help to increase the number of students or junior doctors choosing sports medicine as a future career.

**How it might help clinical practice in the future?**

- Increased teaching and awareness of sports medicine will provide medical students greater opportunities to learn about the specialty which may thus help in their clinical practice skills. Students will feel more confident in regard to the use of sports medicine and physical exercise in the therapy of many different conditions ranging from in a general practice setting to surgical or medical specialties in the future.
