# Supplementary material for: Pilot study: undergraduate sports & exercise medicine conferences: what role do they play?
Source: BMJ Open Sport Exerc Med. 2020 Aug 21;6(1):e000787. doi: 10.1136/bmjsem-2020-000787 (PMC7478070; doi:10.1136/bmjsem-2020-000787)
Supplement: Supplementary data [file bmjsem-2020-000787s001.pdf]

	I have received SEM related teaching during my current degree	I have previously attended a SEM conference or course	I have completed an additional degree, SSC or published project related to SEM	I have had experience of SEM during my current degree
**Yes**	31	13	5	24
**No**	15	33	41	22

Supplementary Table 1. Delegate exposure to Sports & Exercise Medicine (SEM) prior to conference

	Pre-Conference			Post-Conference		
	I was interested in a career in SEM	I understood what a career in SEM involves	I understood what was required to pursue a career in SEM	I am interested in a career in SEM	I understand what a career in SEM involves	I understand what is required of me to pursue a career in SEM
**1-Strongly Agree**	0	3	4	0	0	0
**2-Disagree**	4	7	13	0	0	2
**3-Neutral**	6	7	11	5	9	11
**4-Agree**	24	25	16	25	23	23
**5-Strongly Agree**	12	4	2	16	14	10
**Median Response (IQR)**	4 (4-5)	4 (3-4)	3 (2-4)	4 (4-5)	4 (4-5)	4 (3-4)

Supplementary Table 2. Comparison of understanding and interest in Sports & Exercise Medicine (SEM)

	The content provided during the conference was clear and concise	The speakers engaged the audience	I now feel more knowledgeable about career paths in SEM	I now understand the potential training routes for a career in SEM	Overall I was satisfied with the conference content	I would recommend this conference to a colleague
**1 - Strongly Disagree**	0	0	0	1	0	1
**2 - Disagree**	0	0	1	1	0	0
**3 - Neutral**	2	2	5	13	3	4
**4 - Agree**	28	27	23	25	18	15
**5 - Strongly Agree**	16	17	17	6	25	26
**Median Response (IQR)**	4 (4-5)	4 (4-5)	4 (4-5)	4 (3-4)	5 (4-5)	5 (4-5)

Supplementary Table 3. Evaluation and impact of conference
